# Endobiotic Rugosan Symbionts in Stromatoporoids from the Sheinwoodian (Silurian) of Baltica

**DOI:** 10.1371/journal.pone.0090197

**Published:** 2014-02-25

**Authors:** Olev Vinn, Mari-Ann Mõtus

**Affiliations:** 1 Department of Geology, University of Tartu, Tartu, Estonia; 2 Institute of Geology at Tallinn University of Technology, Tallinn, Estonia; Raymond M. Alf Museum of Paleontology, United States of America

## Abstract

A paleoecological study of stromatoporoid endobionts was carried out to discern the relationships between symbiotic rugosans and their stromatoporoid hosts. The earliest endobiotic rugosan symbiont *Palaeophyllum* sp. in Baltica has only been found in the stromatoporoid *Ecclimadictyon astrolaxum* from Saaremaa, Estonia. The rugosans are vertically oriented inside the stromatoporoid skeleton. Numerous rugosans have their corallites open at the upper, external surface of stromatoporoids, but many are completely embedded within the stromatoporoids. Stromatoporoid hosts were presumably beneficial for rugosans as elevated substrates on a sea floor that offered a higher tier for feeding. Relative substrate stability in the hydrodynamically active shallow waters may have also been beneficial for the rugosans.

## Introduction

In the fossil record, examples of *syn vivo* interactions between different organisms are rather rare. The most common examples comprise predatory borings and endobionts embedded (i.e. bioimmured) by the living tissues of host organisms [1], [2]. Endobiotic invertebrate symbionts appeared in the Late Ordovician (see [3] for review). Fossils of endobionts, both bioclaustrations [4] and shelly fossils, are among the best examples of symbiotic interactions in the fossil record. Bioclaustrations differ from shelly fossils by the lack of their own skeleton. The earliest known skeletal endobiotic symbionts are lingulid brachiopods found in Late Ordovician stromatoporoids of North America [5].

Symbiotic rugosans are well known stromatoporoid endobionts, especially in the Silurian of Baltica [6], [7], [8], [9], [10], [11]. Besides rugosans, the tabulate *Syringopora* is also a common stromatoporoid endobiont in the Silurian of Baltica [9], [12]. The Silurian of Baltica has a rich record of symbiotic endobionts. Recently Vinn and Wilson [13] described a symbiotic cornulitid (*Cornulites stromatoporoides*) and stromatoporoid association from the Abula cliff assemblages. They found 77% of the stromatoporoids studied were infested by cornulitid endobionts.

In addition to stromatoporoids, several other invertebrates host symbiotic endobionts in the Silurian of Baltica. Lingulid brachiopods have been found in borings in tabulate corals of Ludlow age in Gotland [14]. *Chaetosalpinx* bioclaustrations have been documented in tabulate corals of the Llandovery of Estonia [15] and Gotland [16]. *Chaetosalpinx* is a bioclaustration of a worm-like animal with unknown zoological affinities. The aim of this paper is: 1) to describe the earliest known endobiotic rugosan symbionts in stromatoporoids in the Silurian of Baltica and test whether they were host-specific; and 2) to discuss the palaeoecology of the rugosan-stromatoporoid association.

## Geological Background and Locality

During the Silurian, the Baltica paleocontinent was located in an equatorial latitude [17]. The epicontinental Baltica paleobasin in modern Estonia was characterized by a wide range of tropical environments and diverse biotas [18]. On Saaremaa Island, the Silurian succession is represented by shallow shelf carbonate rocks rich in shelly faunas, especially stromatoporoids and tabulates. The Silurian exposures on Saaremaa are mostly small coastal cliffs. Endobiotic rugosans are found in Ludlow strata of the Katri and in Pridoli strata of the Kaugatuma cliffs [9], [11].

**Figure 1:**
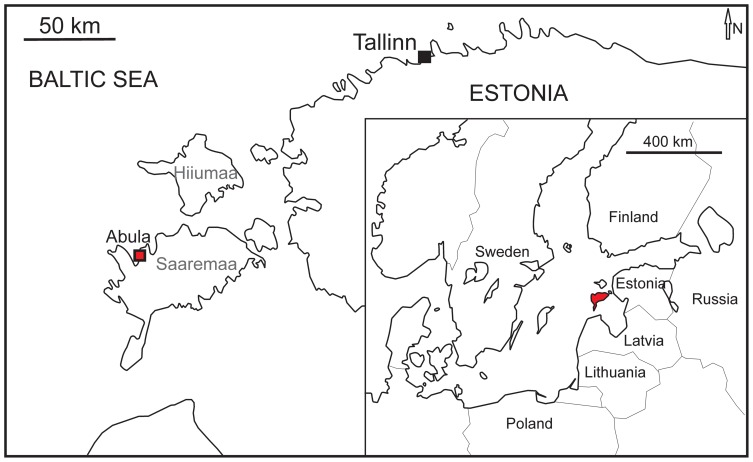
Geographic maps. The location of Abula cliff is shown by square.

**Figure 2:**
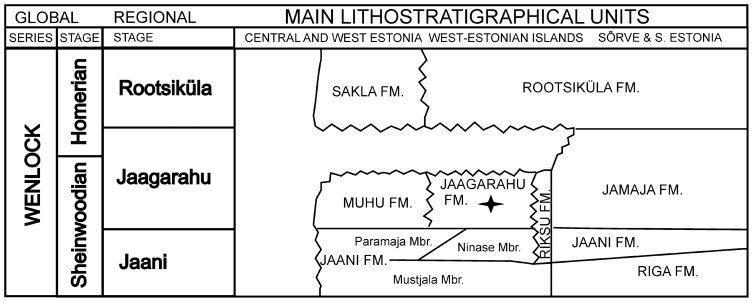
Stratigraphic scheme. The age of Abula fossils is marked by asterisk. Modified after Hints et(2008).

The small Abula cliff is situated on the eastern coast of Tagalaht Bay of Saaremaa about 3 km north of the Mustjala-Veere road (58°27′12′′ N, 22°06′51′′ E) (Figure 1). At the Abula cliff the topmost layers of the Vilsandi Beds (lagoonal dolomitic marlstones) and the basal part of the Maasi Beds are exposed [19]. The Maasi Beds belong to the middle part of the Jaagarahu Formation (Figure 2). The stromatoporoids studied here were collected from the pelletal limestone layers of the Maasi Beds. The stromatoporoid-rich layers are of normal marine origin. They were deposited in shoaling waters of a very shallow sea [20], [21]. Likely wave activity is indicated by the occurrence of rare overturned stromatoporoids, all of which have low domical shapes.

## Materials and Methods

60 stromatoporoids from Abula cliff were searched for endobionts by external observation with magnifying lenses and by breaking them with a hammer. Only 22 stromatoporoid specimens were collected. The collected specimens are deposited at Geological Museum, Museum of Natural History, University of Tartu (TUG). Three stromatoporoid specimens with endobiotic rugosans were cut longitudinally and transversely in the laboratory with a rock saw. Longitudinal and transverse sections were then polished and photographed with a Leica IC80 HD digital camera. Eight thin-sections were made from both transverse and longitudinal sections. All thin-sections were scanned using an Epson 3200 scanner. The areal density of rugosans in the stromatoporoids was estimated using a centimeter grid drawn on a transparent film. All rugosans present in a 4 cm^2^ square were counted.

No permits were required for the described study, which complied with all relevant Estonian regulations, as our study did not involve collecting protected fossil species. Two described stromatoporoid specimens with the endobionts are deposited at Geological Museum, Museum of Natural History, University of Tartu (TUG), Vanemuise 46, Tartu, Estonia, with specimen numbers TUG-1627-4 and TUG-1627-6. One described stromatoporoid specimen with the endobionts is deposited at the Institute of Geology, Tallinn University of Technology (GIT), Ehitajate tee 5, Tallinn, Estonia, with specimen number GIT-666-1.

## Results

**Figure 3:**
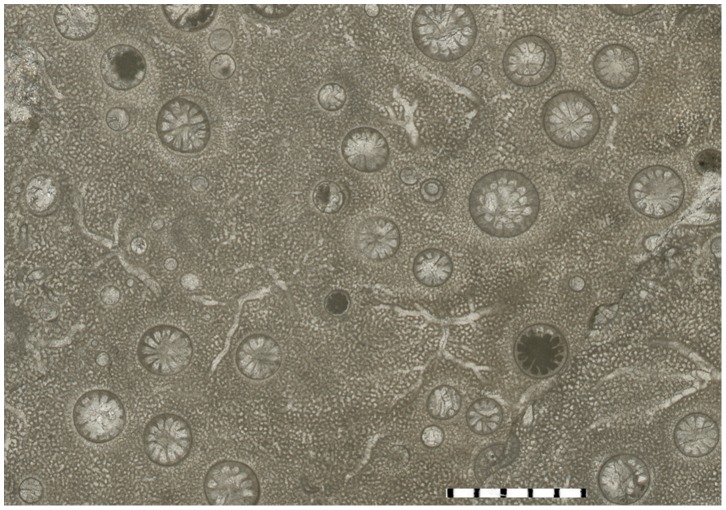
*Palaeophyllum* sp. endobionts. TUG 1627-4. Stromatoporoid *Ecclimadictyon astrolaxum* with the endobionts, transverse section, from Sheinwoodian (Jaagarahu Formation) of Abula cliff, Saaremaa Island, Estonia. Scale bar in mm.

**Figure 4:**
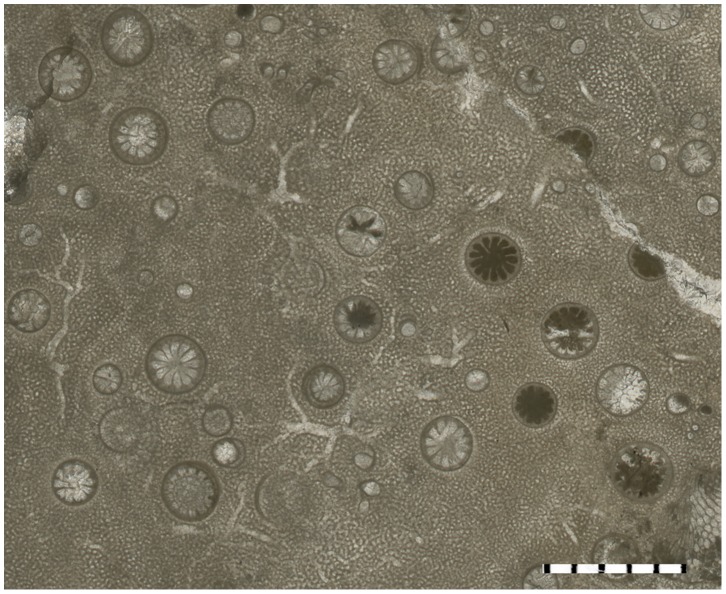
*Palaeophyllum* sp. endobionts. TUG 1627-4. Stromatoporoid *Ecclimadictyon astrolaxum* with the endobionts, transverse section, from Sheinwoodian (Jaagarahu Formation) of Abula cliff, Saaremaa Island, Estonia. Scale bar in mm.

**Figure 5:**
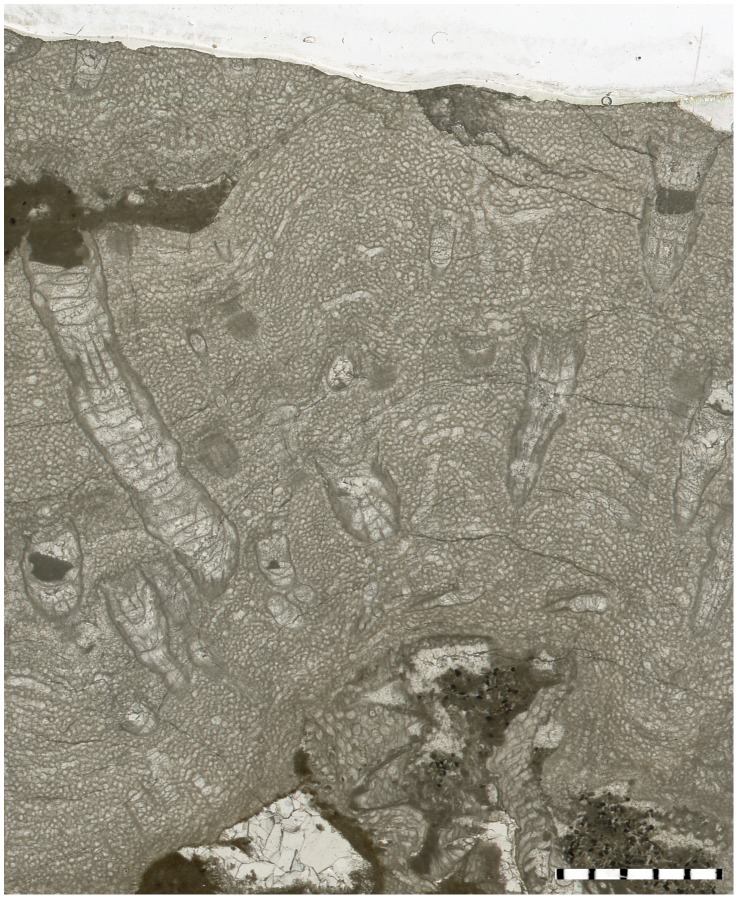
*Palaeophyllum* sp. endobionts. GIT 666-1. Stromatoporoid *Ecclimadictyon astrolaxum* with the endobionts, longitudinal section, from Sheinwoodian (Jaagarahu Formation) of Abula cliff, Saaremaa Island, Estonia. Note completely embedded rugosans and the shaley sediment at the termination of the large rugosan at the left side of the slide. Scale bar in mm.

**Figure 6:**
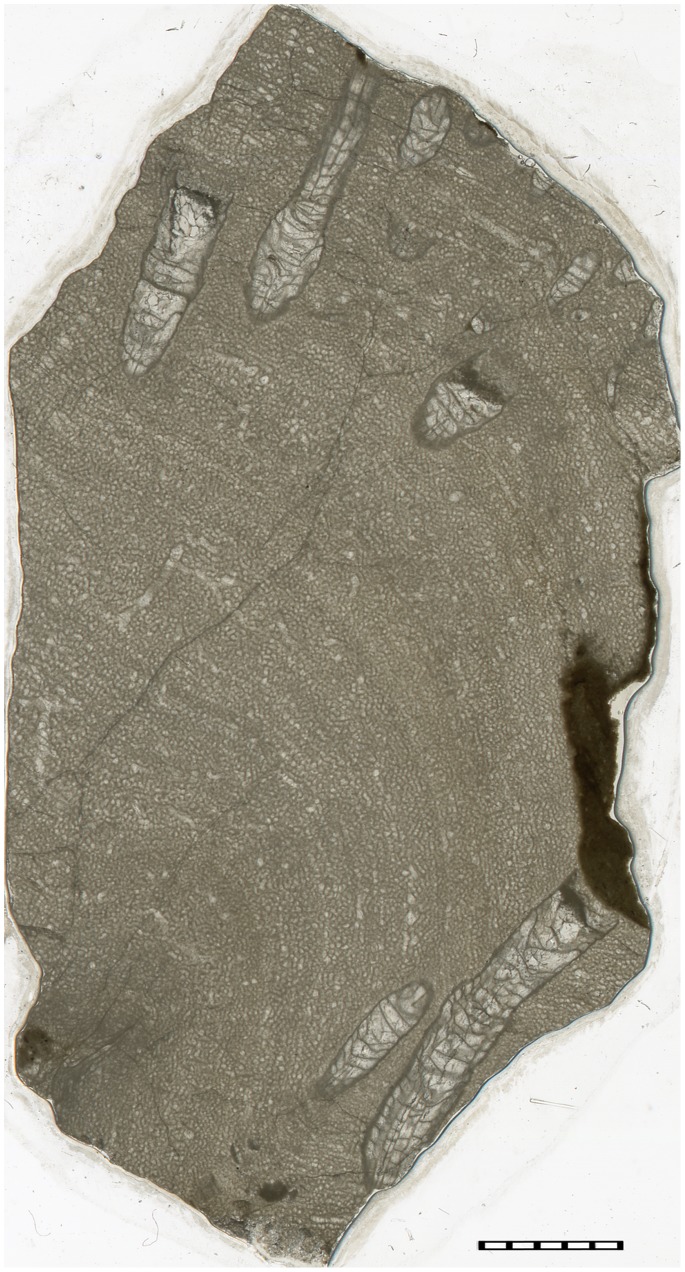
*Palaeophyllum* sp. endobionts. TUG 1627-6. Stromatoporoid *Ecclimadictyon astrolaxum* with the endobionts, longitudinal section, from Sheinwoodian (Jaagarahu Formation) of Abula cliff, Saaremaa Island, Estonia. Note completely embedded rugosans and changing relationship between stromatoporoid laminae and long axis of the rugose coral at the right of the slide. Scale bar in mm.

Only three specimens of 60 examined stromatoporoids contained solitary endobiotic rugosans. The endobiotic rugosans belong to *Palaeophyllum* sp., and they occur only in the stromatoporoid *Ecclimadictyon astrolaxum* Nestor [9]. Specimens of *Palaeophyllum* sp. were found only inside the stromatoporoids (Figures 3–6). Rugosans are perpendicularly oriented to the surface within the stromatoporoid skeleton (Figures 5–6). Numerous rugosans have their corallites opening on the upper external surface of stromatoporoids, but completely embedded rugosans also occur (Figures 5–6). Embedded corallites are distinguished from corallites that diverge from the plane of the section by the lack of rounded terminus. Some corallites are terminated at the level of a dark shaley inclusion of sediment within the stromatoporoid. Rugosans of various apertural diameter occur together on the surface of stromatoporoids (Figures 3–4). The corallites of the rugosans are 0.5 to 3.0 mm wide (mean 1.6 mm, sd = 0.75, N = 15). They are 3.0 to 15 mm long. Some of the embedded tubes (Figures 3–4) with small diameters appear to lack septa, but they also belong to rugosans. All stromatoporoids with endobiotic rugosans have a low domical shape. Ten to 20 endobiotic rugosans were counted per 4 cm^2^. Endobiotic rugosans are usually situated at the level of the surrounding stromatoporoid surface. A few rugosans show slightly elevated corallites (<0.5 mm), and their ends are preserved intact. The rugosan endobionts are spread over the stromatoporoid upper surface without any preference for a particular region and we did not find any relationship to the astrorhizal systems of the stromatoporoids.

## Discussion

### Palaeoecology of the Rugosan-stromatoporoid Association

The *syn vivo* nature of the *Palaeophyllum* sp. and *Ecclimadictyon astrolaxum* association is indicated by orientation of embedded rugosans in the stromatoporoid skeleton. Some divergence of long axes of rugosans (Figure 5) suggests that the orientation of rugosans may have been somewhat environment-controlled. The infested stromatoporoids hosted several generations (i.e., from formation of skeleton until death) of rugosans during their lives, which is indicated by the presence of completely embedded rugosans inside the stromatoporoid skeleton (Figures 5–6). It possible that some apparently truncated corallites actually underwent lateral budding and survived, but we did not find any evidence for that. However, the colonization of the host stromatoporoid by rugosans was a continuous event, because there are no zones of varying infestation density in the vertical sections of the stromatoporoids (i.e., the rugosans are evenly distributed). In addition, rugosans of various corallite diameters occur together on the surface of the host stromatoporoid (Figures 3–4). We did find some possible newly colonizing rugosans tapering to a point in the vertical sections.

It is difficult to determine the exact nature of this symbiotic association using fossil material; everything from mutualism to parasitism may be possible. Stromatoporoid hosts were beneficial for rugosans as elevated substrates on a sea floor that offered a higher tier for feeding. Relative substrate stability in the hydrodynamically active shallow waters may also have been beneficial for the rugosans. The predatory rugosans (microcarnivores) and suspension feeding stromatoporoids (filter feeders) were likely not competitors for food (they may have been feeding on organisms of the same size ranges). However, there may have still been some scramble competition between the rugose corals and their stromatoporoid hosts. Corals might have impoverished the filtrator’s nutrient flow. Similar coral-brachiopod associations have been described, e.g. [22], [23], [24]. The predatory endobiotic rugosans may have even been beneficial for the stromatoporoid host by keeping away small predators. On the other hand, rugosans did occupy some of the feeding surface of the stromatoporoids, which may have been somewhat harmful to the host. Stromatoporoids may have possessed parasites [25], and in some cases corals might have destroyed their host tissues by their sweeper tentacles (like Recent *Platygyra*, see [26] or fossil *Hamarilopora*, see [27]). Similar *syn vivo* rugosan stromatoporoid associations have often been interpreted as commensal [6], [7], [9], [28]. However, most common interactions between different organisms are either mutualistic or parasitic both in the fossil record and the modern biosphere [29], [30].

### Host Selectivity

*Ecclimadictyon macrotuberculatum*, *E. astrolaxum*, *Densastroma pexisum*, and *Vikingia tenuis* are known from the beds [31] exposed at Abula cliff and were collected during the study. *Vikingia tenuis* is the most common stromatoporoid species at the studied locality. Thus, it is possible that the symbiotic rugosan-stromatoporoid association described here occurred only or dominantly between *Palaeophyllum* sp. and *Ecclimadictyon astrolaxum*. This could be explained by the greater tolerance of *Ecclimadictyon astrolaxum* for infesting rugosans, because it does not seem feasible that rugosans would have benefited from infesting only this particular stromatoporoid species. Endobiotic *Palaeophyllum* sp. has been described from *Plectostroma intermedium* from Ludlow of Gotland [9]. Thus *Palaeophyllum*, but probably a different species, also inhabited other stromatoporoid species. Most rugosans infested more than one stromatoporoid species in the Ludlow of Gotland [9]. The only specimens of *Palaeophyllum* sp. from Abula cliff are found inside the stromatoporoid *Ecclimadictyon astrolaxum*. The coral species has not been described from here or any other locality living independently of stromatoporoids. Further studies should show whether this rugosan-stromatoporoid symbiotic relationship was obligatory or facultative for the coral.

### Distribution of Symbiotic Associations Involving Rugose Corals

Stromatoporoid-rugosan associations similar to the Abula one are known from the Silurian of Gotland [6]–[9] and the Devonian of Spain [28]. An endobiotic rugosan symbiont in a stromatoporoid has recently been described from the Pridoli of Saaremaa, Estonia [11]. Discovery of symbiotic rugosans in the stromatoporoids from Abula cliff could indicate that symbiotic rugosans may have been more common in the Silurian of Baltica than previously known.

In addition to stromatoporoids, endobiotic rugosans can form symbiotic associations with tabulate corals and crinoids. An endobiotic rugosan coral *Streptelasma* sp. has been described in the tabulate coral *Paleofavosites prolificus* from the Llandovery of Ohio [32]. In the Devonian of Morocco and Germany, rugose corals infested the stems of living crinoids [33], [34]. However, data from the literature and this described association show that stromatoporoids were the commonest fossilized host organisms for endobiotic rugosans in the Palaeozoic.
